# CRISPR deletion of a SINE-VNTR-*Alu* (SVA_67) retrotransposon demonstrates its ability to differentially modulate gene expression at the *MAPT* locus

**DOI:** 10.3389/fneur.2023.1273036

**Published:** 2023-09-29

**Authors:** Alexander Fröhlich, Lauren S. Hughes, Ben Middlehurst, Abigail L. Pfaff, Vivien J. Bubb, Sulev Koks, John P. Quinn

**Affiliations:** ^1^Department of Pharmacology and Therapeutics, Institute of Systems, Molecular and Integrative Biology, University of Liverpool, Liverpool, United Kingdom; ^2^Perron Institute for Neurological and Translational Science, Perth, WA, Australia; ^3^Centre for Molecular Medicine and Innovative Therapeutics, Murdoch University, Perth, WA, Australia

**Keywords:** SINE-VNTR-*Alu*, retrotransposon, CRISPR, gene regulation, gene expression, Parkinson’s disease, *MAPT* locus

## Abstract

**Background:**

SINE-VNTR-*Alu* (SVA) retrotransposons are hominid-specific elements which have been shown to play important roles in processes such as chromatin structure remodelling and regulation of gene expression demonstrating that these repetitive elements exert regulatory functions. We have previously shown that the presence or absence of a specific SVA element, termed SVA_67, was associated with differential expression of several genes at the *MAPT* locus, a locus associated with Parkinson’s Disease (PD) and frontotemporal dementia. However, we were not able to demonstrate that causation of differential gene expression was directed by the SVA due to lack of functional validation.

**Methods:**

We performed CRISPR to delete SVA_67 in the HEK293 cell line. Quantification of target gene expression was performed using qPCR to assess the effects on expression in response to the deletion of SVA_67. Differences between CRISPR edit and control cell lines were analysed using two-tailed t-test with a minimum 95% confidence interval to determine statistical significance.

**Results:**

In this study, we provide data highlighting the SVA-specific effect on differential gene expression. We demonstrate that the hemizygous deletion of the endogenous SVA_67 in CRISPR edited cell lines was associated with differential expression of several genes at the *MAPT* locus associated with neurodegenerative diseases including *KANSL1*, *MAPT* and *LRRC37A*.

**Discussion:**

This data is consistent with our previous bioinformatic work of differential gene expression analysis using transcriptomic data from the Parkinson’s Progression Markers Initiative (PPMI) cohort. As SVAs have regulatory influences on gene expression, and insertion polymorphisms contribute to interpersonal differences in expression patterns, these results highlight the potential contribution of these elements to complex diseases with potentially many genetic components, such as PD.

## 1. Introduction

Neurodegenerative diseases, including Parkinson’s disease (PD) and frontotemporal dementia (FTD), affect millions of people worldwide. PD is characterised by the loss of dopaminergic neurons in the substantia nigra and the presence of Lewy bodies leading to a lack of dopamine supplied to the brain (1). Characteristic symptoms include resting tremors, bradykinesia and other motor and non-motor manifestations (2). In the effort to explore potential PD-related genetic factors, several large-scale genome-wide association studies (GWAS) analysing PD-associated single nucleotide polymorphisms (SNPs) have been performed (3–6). In one of the most comprehensive GWAS to date, Nalls et al. (4) identified 90 PD-associated risk signals across the genome, however, these signals only explained 16%–36% of heritable PD risk. FTD, which involves death of cortical neurons in the frontal and temporal lobes, accounts for 3%–26% of dementia cases and is therefore the most common presenile dementia after Alzheimer’s disease (7, 8). FTD can also be diagnosed in combination with amyotrophic lateral sclerosis (ALS) leading to the disease spectrum ALS-FTD (9). Approximately 50% of ALS patients suffer from cognitive impairment, while up to 15% manifest symptoms of FTD which include disturbances of behaviour, personality, and language (8, 9). In FTD, it is estimated that 10%–30% of the cases are inherited in an autosomal-dominant fashion, with *C9ORF72* (20%–30%) and *MAPT* (5%–20%) variations being the most common (10). However, to date for both PD and FTD a large proportion of the contributing genetics is unexplained, frequently known as “missing heritability.” Further investigation into the potential factors contributing to the missing heritability is critical, with one area of interest being transposable elements (TEs).

TEs constitute around 45% of the human genome and are divided into two families, the DNA transposons and the retrotransposons (11–15). A subset of elements belonging to the latter class still remain active in the human genome (16), this includes the long interspersed nuclear elements (LINEs), *Alu*, and SINE-VNTR-*Alu* (SVA) elements, which have demonstrated not only to be drivers of genetic diversity by contributing to genome variation, gene regulation and hominid evolution (17–19) but also to be involved in disease development and progression (16, 20–22). Genetic diversity including the capacity of TEs to act as transcriptional regulators can be achieved by a variety of mechanisms, for instance, by providing alternative splice sites, polyadenylation signals and promoters whilst harbouring sites for transcription factor (TF) binding (23–27).

**Figure 1:**
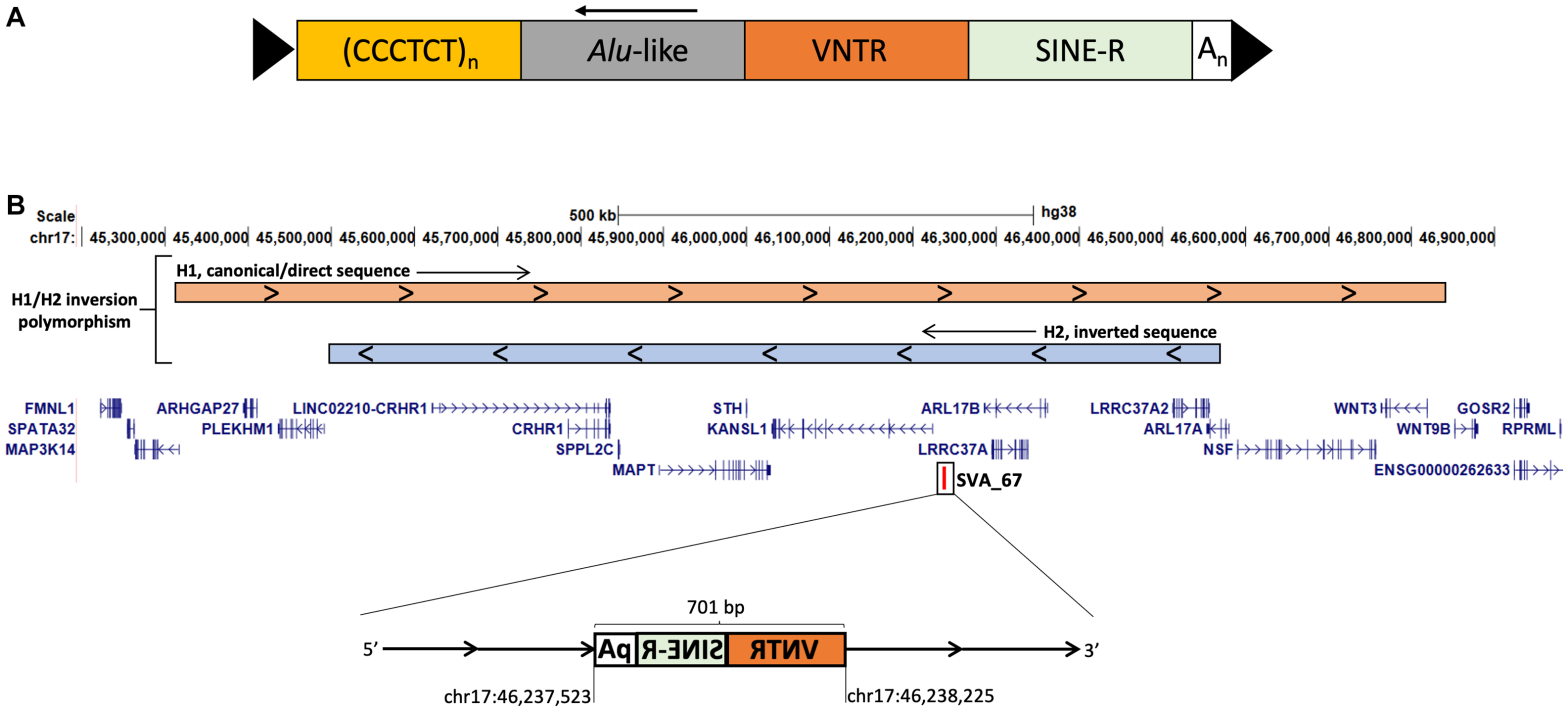
SVA structure and schematic representation of the *MAPT* locus on chromosome 17 modified from UCSC genome browser hg38. **(A)** Full-length SVA elements contain a 5′ CT element, *Alu*-like region, GC-rich VNTR (variable number tandem repeat), SINE (short interspersed nuclear element)-R domain and a 3′ poly-A tail flanked by target site duplications (TSDs). **(B)** The *MAPT* locus is characterised by an inversion polymorphism called *H1*/*H2*. *H2* is characterised by a ~970 kb inversion compared to H1 the canonical sequence (visualised at the top). The truncated SVA element, SVA_67 (chr17:46,237,523–46,238,225) represents a 701 bp SVA element and is located approximately 12 kb upstream and in sense orientation with reference to the 5′ transcriptional start site of *KANSL1*. SVA_67 is present in *H1* and absent in *H2*. Start and end coordinates of SVA_67 are shown.

SVA elements belong to the non-LTR (long terminal repeat) retrotransposon family, they are typically 0.7–4 kb in length and there have been around 3,000 SVAs identified in the reference human genome (28–32). Full-length SVA elements contain a 5’ CT element, *Alu*-like region, GC-rich VNTR (variable number tandem repeat), SINE (short interspersed nuclear element)-R domain and a 3′ poly-A tail (Figure 1A) (33). SVAs can be polymorphic for both presence/absence within the hominid genome referred to as retrotransposon insertion polymorphisms (RIPs) and in structure (22, 34). Polymorphisms within SVA structure and SVA presence or absence add additional layers of complexity to gene expression dynamics and could be associated with predisposition to disease, for example previously we have identified 81 SVA RIPs in the Parkinson’s progression markers initiative (PPMI) cohort, with a small subset significantly associated with PD progression and differential gene expression (22). Interestingly, one of these RIPs represents SVA_67, a truncated SVA element, which lies within the microtubule-associated protein tau (*MAPT*) locus on chromosome 17q21.31 (Figure 1B), a locus which has been shown to contribute to increased risk of PD and FTD (35, 36). The *MAPT* locus itself includes two predominant haplotypes, termed *H1* and *H2*, whereby the *H2* haplotype is characterised by a ~970 kb inversion polymorphism and also lacks SVA_67, compared to the SVA_67 containing *H1* haplotype (36, 37). The *H1/H2* polymorphism is primarily found in the Mediterranean with ranges within Europe between 5% and 37.5% (38). We have previously demonstrated that SVA_67 presence or absence was significantly associated with differential expression of several genes at the *MAPT* locus, including genes associated with FTD or PD including *MAPT*, *KANSL1* and *LRRC37A* (22, 34). However, we were not able to demonstrate that causation of differential gene expression was directed by the SVA due to lack of functional validation. In order to investigate the functional significance of SVA_67 within the *H1* haplotype and its potential contribution to disease mechanisms, we aimed to validate previous findings by applying CRISPR to excise SVA_67 and compare gene expression patterns in an otherwise identical genetic background. This study expands the analysis of SVA_67 activity as a regulatory domain hypothesised to lead to interpersonal expression patterns and may help to better understand the involvement of SVA_67 within neurodegenerative diseases.

## 2. Materials and methods

### 2.1. CRISPR deletion of SVA_67 in HEK293 cell line

The CRISPR (clustered regularly interspaced short palindromic repeats) method applied in this study was adapted from Ran et al. (39) using a non-homologous end joining approach. The EF1α-pSpCas9(BB)-2A-GFP plasmid used provided both the Cas9 machinery and associated gRNA with RNA scaffold necessary for precise editing of a genomic site. For targeted deletion of SVA_67, two CRISPR constructs were generated by golden gate cloning containing guide RNAs (gRNAs) targeting specific sequences upstream and downstream, respectively, of SVA_67. The gRNAs were as follows and their successful integration into the plasmid was confirmed by sequencing:

1. Upstream: Fw1 5′-CACAGCTGTTCGGGAGACTG-3′ (chr17:46,237,413-46,237,432).
2. Downstream: Rv1 5′-TCGAGACTAACCTGACCGGT-3′ (chr17:46,238,223-46,238,242).

For transfection, 100,000 HEK293 cells (obtained from ATCC, CRL-1573) were co-transfected with both pSpCas9(BB)-2A-GFP containing gRNA constructs using Turbofect transfection reagent (Thermo Fisher) according to manufacturer’s instructions. Forty-eight hours post transfection, the cells were dissociated to obtain a single cell suspension and seeded at low density (1,000 cells per 10 cm petri dish). Cells were cultured until single colonies of clonal cell populations were seen and then transferred into two 96-well plates, whereby one continued in culture and the other was processed for genotyping PCR. The deletion of SVA_67 was assessed by PCR using the primers SVA_67_Fw (5′-AGTTGACCGTAATGTGAGCACT-3′) and SVA_67_Rv (5′-AGCCCCACAGGTAATACTTAATGA-3′) in the following reaction and reagents (final concentration): KOD hot start buffer (1×, Merck), MgSO_4_ (1.5 mM, Merck), dNTPs (0.2 mM each, Merck), betaine (1 M, Sigma), Primer SVA_67_Fw and SVA_67_Rv (0.3 μM each, Sigma), KOD hot start DNA polymerase (0.02 U/μL), made up with nuclease-free water to a final volume of 20 μL. Amplification reactions were performed using the SimpliAmp^™^ Thermal Cycler (Applied Biosystems) and the following programme: 95°C for 2 min, 95°C for 20 s, 62°C for 10 s and 70°C for 50 s, repeating steps 2–4 34 more times. This PCR resulted in either a 2,795/2,566 bp product when SVA_67 was present (two different SVA_67 alleles are present in HEK293 cells) or 1,734 bp after deletion of SVA_67.

### 2.2. qPCR of target gene expression in CRISPR edited cell lines

Quantification of target gene expression was performed using qPCR to assess the effects on expression in response to the knockout of SVA_67. Generated HEK293 SVA_67 CRISPR control (unedited) and SVA_67 CRISPR edited cell lines were utilised to assess differential gene expression of five target genes at the *MAPT* locus (*KANSL1*, *MAPT*, *ARL17A, ARL17B* and *LRRC37A*). To generate cDNA from three CRISPR control and three CRISPR edit clonal cell lines for qPCR experiments, total RNA was extracted using the Monarch RNA Miniprep Kit (New England Biolabs). The GoScript^™^ Reverse Transcription System (Promega) was used for first-strand cDNA synthesis from 3 μg total RNA according to manufacturer’s instructions. The qPCR cycling conditions and primer sequences are shown in Table 1. For each experiment, a *β-actin* qPCR was included as reference gene alongside each target gene qPCR. Reactions were performed in three biological replicates (with three technical replicates in each reaction) for the CRISPR control and CRISPR edited cell lines, per gene. An AriaMX real-time PCR system (Agilent) was used to analyse gene expression by collecting quantification cycle (*C*_q_) values. Analysis was performed using the Agilent Aria 1.8 software. Target gene expression was quantified using the 2^−ΔΔCT^ method. Differences between CRISPR edit and control cell lines were analysed using two-tailed *t*-test with a minimum 95% confidence interval to determine statistical significance. Generated amplification and melting curves as well as primer efficiencies can be found in Supplementary Figure S1.

**Table 1:** Primer sequences and cycling condition for qPCR analysis.

Target gene	Sequence	Amplicon size (bp)	Cycling condition
*KANSL1*	F: 5′-ATCCTCCACACAGTCCCTTG-3′ R: 5′-CCCCTTCTCCTCCTTACTGG-3′	121	(1 cycle): 95°C – 3 m (40 cycles): 95°C – 15 s 60°C – 1 m (Melting curve, 1 cycle): 95°C –1 m 55°C – 30 s 95°C – 30 s
*ARL17A*	F: 5′-AGACTACAGGTGCATACTAC-3′ R: 5′-CTTAAATTACATGGGGCCAG-3′	146	
*MAPT*	F: 5′-CGTCCCTGGCGGAGGAAATA-3′ R: 5′-CCCGTGGTCTGTCTTGGCTT-3′	82	
*ARL17B*	F: 5′-TACAGTAGGTTTCTGTGTGG-3′ R: 5′-GAGGTCTGATTTTGAAGTGG-3′	89	
*LRRC37A*	F: 5′-GTTGTCACCGAAGGTCATTACAAG-3′ R: 5′-GAGGAAAGTCCATCCTGACTC-3′	115	
*β-actin*	F: 5′-GATCAAGATCATTGCTCCTC-3′ R: 5′-TTGTCAAGAAAGGGTGTAAC-3′	191	

## 3. Results

### 3.1. CRISPR deletion of SVA_67 in HEK293 cell line

Our previous studies indicated that SVA_67 presence or absence correlated with differential gene expression of multiple genes at the *MAPT* locus (22, 34). To validate if this was associated with a potential regulatory activity of SVA_67, we performed CRISPR in HEK293 cells followed by qPCR analysis for target genes at the *MAPT* locus. The SVA_67 primary sequence is polymorphic in the VNTR domain (Fröhlich et al., 2023, submitted) in the HEK293 cell line, two amplicon sizes were obtained when amplifying over the SVA_67 element, namely 2,795 and 2,566 bp.

**Figure 2:**
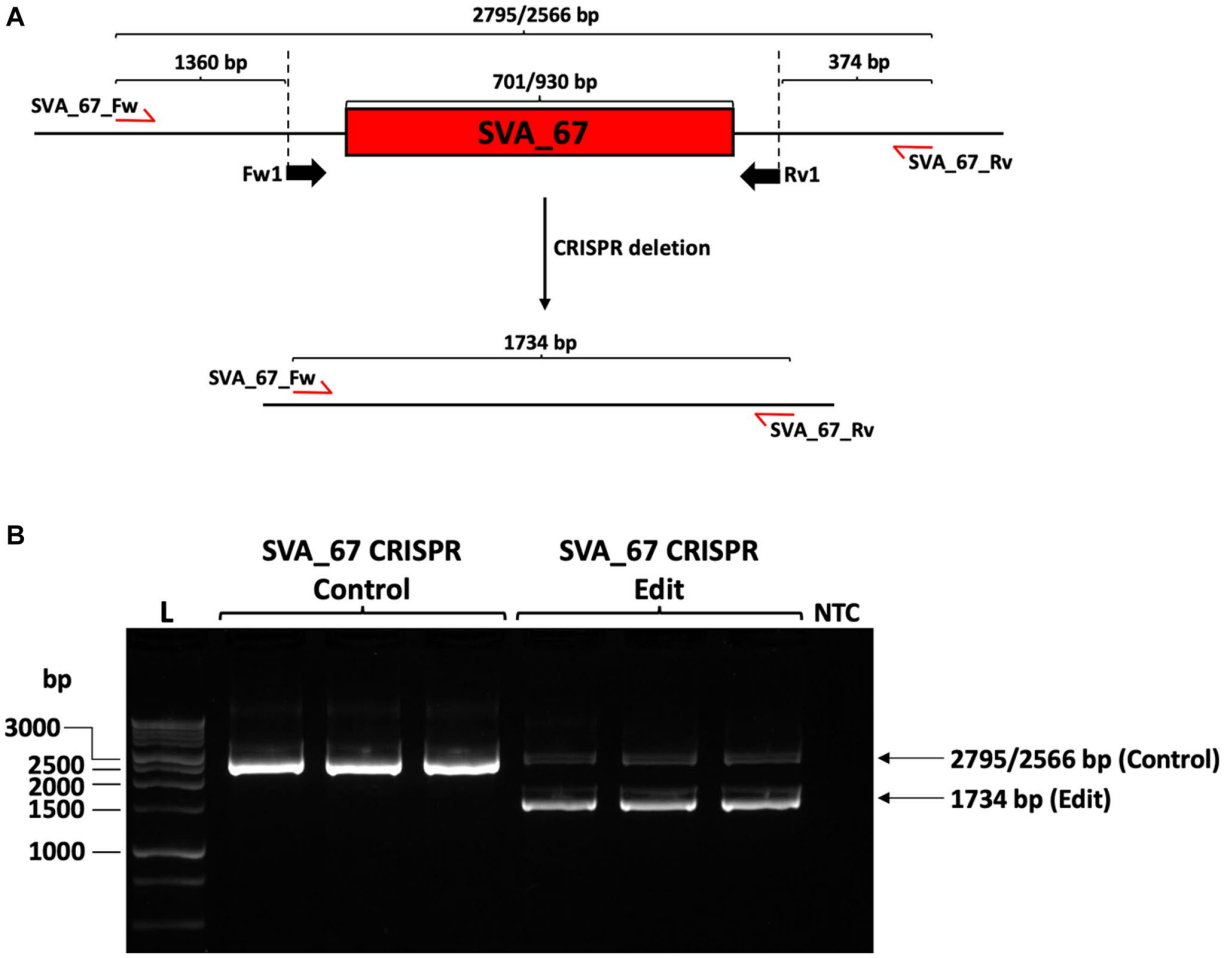
CRISPR deletion of SVA_67 in HEK293 cell line. **(A)** HEK293 cell line is characterised by a primary sequence polymorphism of SVA_67 with two different SVA_67 alleles (701/930 bp) present. To delete SVA_67, one forward (Fw1) and one reverse (Rv1) guide RNA flanking SVA_67 were designed, cloned into EF1α-pSpCas9(BB)-2A-GFP plasmid and transfected into HEK293 cells. Predicted PCR amplicon sizes upon deletion are indicated. Red arrows show the approximate position of the PCR primers for subsequent modification PCR analysis. **(B)** PCR amplification with primer set SVA_67_Fw and SVA_67_Rv was performed using gDNA preparations isolated from HEK293 CRISPR cell populations. Representative gel image with expected amplicon sizes with SVA_67 presence or absence is shown indicating that three SVA_67 CRISPR control and edited cell lines were generated. NTC, non-template control.

Two guide RNA molecules were designed that cut upstream and downstream, respectively, of SVA_67, thereby initiating the excision by induction of double-stranded breaks followed by non-homologous end-joining which would result in a PCR amplicon of 1,734 bp upon successful excision (Figure 2A). PCR amplification confirmed the hemizygous deletion of SVA_67 in HEK293 cell clonal populations by obtaining the corresponding PCR amplicon size of 1,734 bp (Figure 2B). However, a complete SVA_67 knock-out (KO) cell line (homozygous absent) was not obtained, reflected by the continued presence of amplicons of 2,795 and 2,566 bp in length (Figure 2B). In total, three clonal HEK293 cell populations were obtained where an allele of SVA_67 has been deleted (SVA_67 CRISPR edit). For comparison, three control cell lines (SVA_67 CRISPR control) were also selected from the transfected batch which had undergone the CRISPR procedure but did not have a detectable deletion by PCR.

### 3.2. CRISPR deletion of SVA_67 is significantly associated with differential gene expression

**Figure 3:**
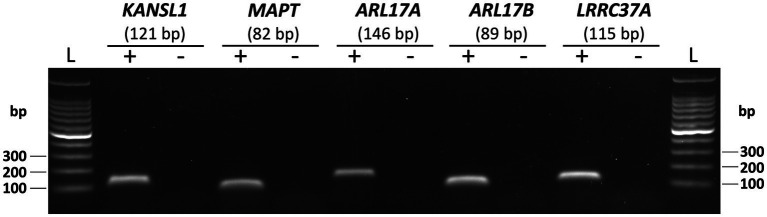
Endogenous expression of five genes at the *MAPT* locus in HEK293 cell line. PCR with primer sets targeting *KANSL1*, *MAPT*, *ARL17A*, *ARL17B* and *LRRC37A* was performed using HEK293 cDNA as template (+). Representative gel image with expected amplicon sizes for each gene is shown. Non-template controls (−) with no cDNA as template for PCR were included for each gene.

Having demonstrated the hemizygous deletion of SVA_67 in a HEK293 cell line model, we next aimed to analyse gene expression changes upon excision of SVA_67. For analysis, we chose five genes (*KANSL1*, *MAPT*, *ARL17A*, *ARL17B* and *LRRC37A*) whose expression has previously shown to be significantly affected by presence or absence of SVA_67 in the PPMI cohort (22). Firstly, we confirmed that the five genes chosen were endogenously expressed in the HEK cell line. Corresponding PCR amplicons of 121 bp (*KANSL1*), 82 bp (*MAPT*), 146 bp (*ARL17A*), 89 bp (*ARL17B*) and 115 bp (*LRRC37A*) were obtained confirming expression of these genes in the chosen cell line (Figure 3).

**Figure 4:**
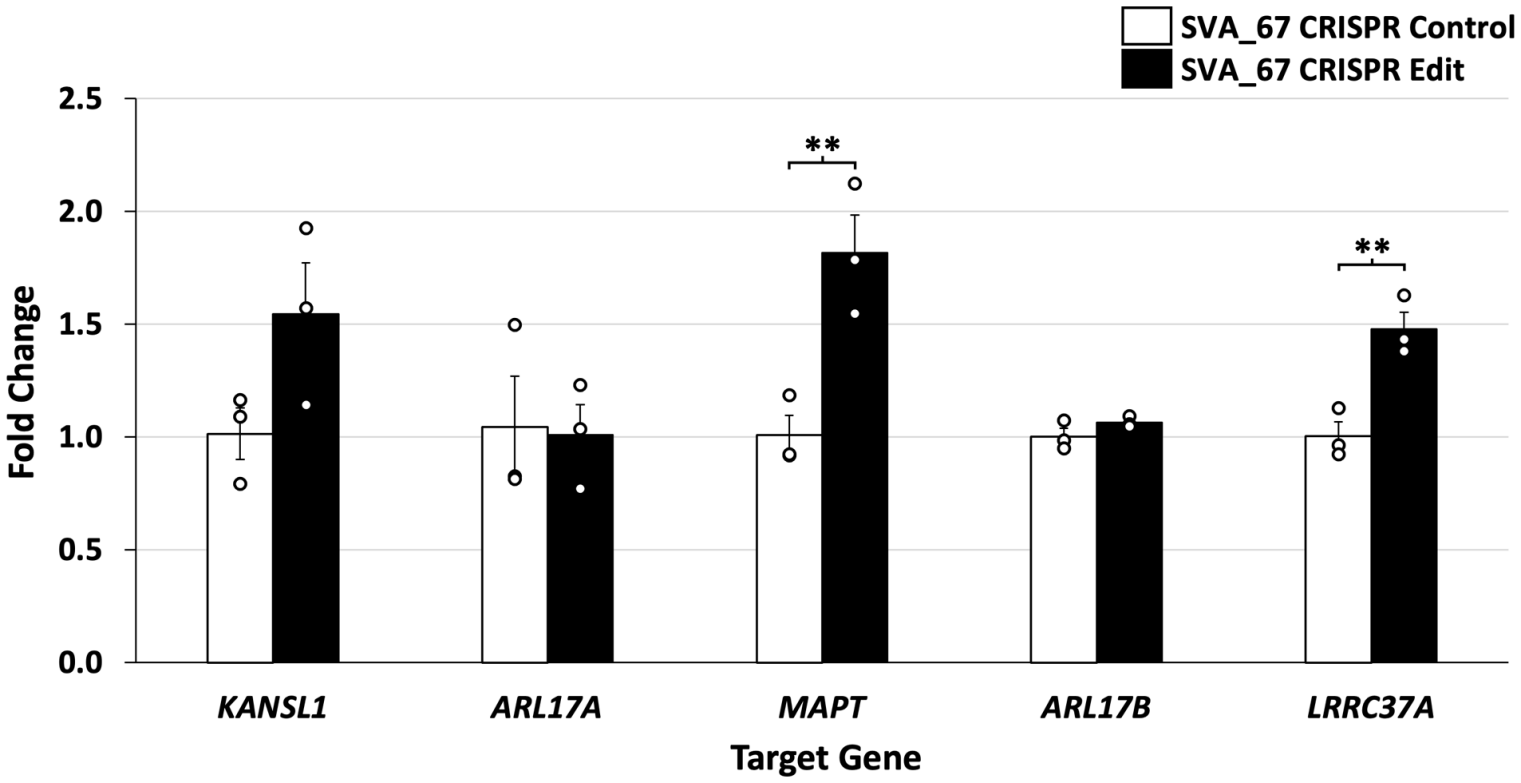
Deletion of SVA_67 is significantly associated with differential gene expression in HEK293 CRISPR cell lines. Quantitative PCR (qPCR) of five genes (*KANSL1*, *ARL17A*, *MAPT*, *ARL17B* and *LRRC37A*) located at the *MAPT* locus was performed. Data analysis showed increased gene expression for *KANSL1* (50%), *MAPT* (82%) and *LRRC37A* (48%) in SVA_67 CRISPR cell lines compared to controls. Fold change in expression was calculated relative to a CRISPR SVA_67 control cell line (fold change = 1) and normalised to *β-actin* expression using the ΔΔCT method. Each qPCR experiment was performed with three biological replicates *N* = 3 and technical replicates *N* = 3. Difference between CRISPR edit and control was analysed using two-tailed *t*-test with a minimum 95% confidence interval to determine statistical significance. Error bars set by standard error mean. ^**^p < 0.01.

Expression of these target genes was assessed by qPCR using cDNA generated from HEK293 CRISPR SVA_67 control and edit cell lines. Gene expression analysis showed that hemizygous deletion of SVA_67 was significantly associated with increased expression of *MAPT* and *LRRC37A* (Figure 4), namely increases in expression of 82% for *MAPT* (*p* = 0.0092) and 48% for *LRRC37A* (*p* = 0.0078). In addition, a 50% increase in *KANSL1* gene expression correlated with the SVA_67 CRISPR edit cell lines compared to controls, however this did not reach statistical significance (*p* = 0.0999). No association was detected for *ARL17A* and *ARL17B* (Figure 4). We compared this data to our previous work analysing the effect of SVA_67 allele dosage on differential gene expression using transcriptomic data from the PPMI cohort (22). Within this cohort, individuals who had only one SVA_67 allele (PA) showed a 20% increase in expression for *KANSL1* and *LRRC37A* compared to individuals who had two copies of this SVA present in the genome, while for *MAPT* the opposite effect was detected (20% decrease in heterozygous/PA individuals (Table 2). No significant change was detected for *ARL17B* which is consistent with this study (Table 2).

**Table 2:** Comparison of gene expression analysis.

Target gene	qPCR fold change CRISPR edit vs. control cell lines	Transcriptomic data fold change PA vs. PP from Pfaff et al. (22)
*KANSL1*	1.50	1.20
*MAPT*	1.82	0.80
*LRRC37A*	1.48	1.20
*ARL17A*	1.01	1.60
*ARL17B*	1.06	0.85

Fold change increase/decrease for genes in the SVA_67 CRISPR edited cell lines relative to the SVA_67 CRISPR control is shown. Data was compared to bioinformatic analysis from Pfaff et al. (22) showing fold change of individuals having one allele of SVA_67 present (heterozygous/PA genotype) compared to both alleles (homozygous present/PP).

## 4. Discussion

Previous studies have demonstrated associations between the *MAPT* locus *H1* haplotype and PD and FTD risk (35, 36), however the specific functional variant remains undetermined. We have previously shown through bioinformatic analysis that SVA_67 presence or absence correlated with differential gene expression and progression of PD using clinical and transcriptomic data from the Parkinson’s progression markers initiative (PPMI) cohort (22, 34). In this study, we expanded and validated previous findings by demonstrating that the hemizygous deletion of SVA_67 in CRISPR-edited cell lines was associated with differential gene expression of *MAPT*, *KANSL1* and *LRRC37A* (Figure 4). This not only highlights the plethora of transcriptomic changes associated with SVA_67 insertion polymorphism but also enables further insight into the role of SVAs within disease pathology and progression.

Through the incorporation of SVA_67 CRISPR-edited HEK293 cell lines in qPCR analysis, we demonstrated that upon hemizygous deletion of SVA_67, differential gene expression was observed, whereby *KANSL1, MAPT* and *LRRC37A* displayed a 50% (1.5-fold), 82% (1.82-fold) and 48% (1.48-fold), increase respectively, in expression compared to unedited control cells (Figure 4). These findings are consistent with our previous work, where we analysed the effect of SVA_67 allele dosage on differential gene expression using transcriptomic data from the PPMI cohort (Table 2) (22). Within this cohort, individuals who had only one SVA_67 allele (PA) showed a 1.2-fold increase in expression for *KANSL1* and *LRRC37A* compared to individuals who had two copies of this SVA present in the genome (22). We do appreciate the complex karyotype of HEK293 cells used in this study which results in a certain limitation to fully compare both datasets, however the SVA_67 CRISPR edited cell model with hemizygous deletion of this SVA is closest to the heterozygous genotypes from PPMI individuals. Another limitation of this study represents the used HEK293 cell line. Although this cell line has previously shown to manifest markers of neuronal cells, it is not the most suitable cell line to assess the involvement of transposable elements within PD and FTD genetics. However, this cell line provided insights into the regulatory mechanisms associated with TE polymorphism and the results were supported by *in vivo* patient-derived transcriptomic data.

The affected genes *KANSL1* and *LRRC37A* have previously been shown to be associated with PD. The PD GWAS risk gene candidate *KANSL1* is part of the non-specific lethal (NSL) complex and has been identified as an essential gene for autophagy, whereby impaired autophagy has been considered to be a potential cause for neurodegenerative diseases such as PD. (40) Additionally, Soutar et al. (41) identified *KANSL1* as a regulator of PINK1 (PTEN-induced kinase 1)-mitophagy in idiopathic PD. They showed that *KANSL1* knockdown led to a reduction in phosphorylation of ubiquitin [pUb(Ser65)], a PINK1-dependant mitophagy initiation marker, and a reduction in expression of PINK-1. The authors proposed that *KANSL1* may be the driver of PD risk at the locus as the knock down of 30 other genes from the locus including *MAPT* did not replicate this disruption of ubiquitin phosphorylation. Along with this, RNA-seq analysis by Bowles et al. (42) investigating *LRRC37A* has recently demonstrated localisation of LRRC37A to human substantia nigra tissue, specifically astrocytes, the dysfunction of which has been considered to be involved in PD pathogenesis. This study showed that LRRC37A is associated with PD via both its involvement in astroglial inflammation regulation, and interaction and co-localisation with α-synuclein.

A 0.8-fold decrease in expression for *MAPT* (encoding tau protein) in PA individuals compared to PP was detected in our previous study, however our qPCR results demonstrated a 1.82-fold increase in expression (Figure 4 and Table 2). It can be argued that these differences are not to be unexpected when comparing cell line data and human blood transcriptomic data, as we focus on the role of SVA_67 in modulating expression in conjunction with other regulatory domains and signals varying in the cells analysed. Aggregation of tau is a hallmark of many neurodegenerative diseases (e.g., Alzheimer’s disease), however there is accumulating evidence for tau pathology in PD brains (43).

Overall, this study highlights SVA_67 as a regulatory domain to modulate target gene expression at the *MAPT* locus. This would be consistent with the SVA’s ability to bind CCCTC-binding factor (CTCF). This protein possesses a key role in 3D chromatin regulation and looping through the cooperation with protein complex cohesion which brings regulatory elements and promoters together in 3D space to facilitate or repress gene expression (44, 45). To silence/control expression of retrotransposons, host defence mechanisms have been established involving the modification of the epigenetic landscape in form of histone modification and DNA methylation (46). Within this mechanism, Kruppel associated box (KRAB) zinc finger proteins (ZFPs) play a major role by binding sequences of retrotransposons, thereby recruiting tripartite motif-containing 28 (TRIM28), also known as KRAB-associated protein 1 (KAP1) which elicits heterochromatin formation (compact state) and transcriptional silencing through NuRD-mediated histone deacetylation and SetDB1 methylation (47). This interaction involving retrotransposon activity and its suppression by epigenetic mechanisms has led to a so called “evolutionary arms race” (18). These epigenetic mechanisms can also be associated with regulation of genes located around the specific retrotransposon of interest. One example involved KO of KAP1 in murine neural progenitor cell models which correlated with a reduced TE epigenetic silencing. Based on this, an increased expression of genes located in proximity (<50 kb) to endogenous retrovirus elements was detected in *Trim28*^−/−^ cells (48). In the present study, it was shown that KO of SVA_67 led to increased expression of *KANSL1* and *LRRC37A* (Figure 4), both genes located <60 kb away from SVA_67 which is located between these two genes. Future work can address coverage not only of CTCF but also H3K9me3 over SVA_67 using chromatin immunoprecipitation (ChIP) with a PCR-based approach, whilst also making use of Oxford Nanopore Technologies (ONT) to characterise methylation patterns across the *MAPT* locus to support suggested mode of actions of SVA_67. Ultimately, this work shows that SVA_67 demonstrates a significant impact on expression of genes associated with neurodegenerative diseases like PD or FTD. The ability of SVA_67 to act as a regulatory domain and contributor to interpersonal expression patterns could raise an appreciation of the involvement of TEs in the missing heritability of neurodegenerative diseases.

## Data availability statement

The original contributions presented in the study are included in the article/Supplementary material, further inquiries can be directed to the corresponding author.

## Author contributions

AF: Conceptualization, Methodology, Writing – original draft, Writing – review & editing. LH: Conceptualization, Methodology, Writing – review & editing. BM: Conceptualization, Funding acquisition, Methodology, Writing – review & editing. AP: Conceptualization, Funding acquisition, Writing – review & editing. VB: Conceptualization, Funding acquisition, Methodology, Writing – review & editing. SK: Conceptualization, Funding acquisition, Writing – review & editing. JQ: Conceptualization, Funding acquisition, Methodology, Writing – review & editing.
