# Supplementary material for: CRISPR deletion of a SINE-VNTR-Alu (SVA_67) retrotransposon demonstrates its ability to differentially modulate gene expression at the MAPT locus
Source: Front Neurol. 2023 Sep 29;14:1273036. doi: 10.3389/fneur.2023.1273036 (PMC10570551; doi:10.3389/fneur.2023.1273036)
Supplement: Supplementary file 1 [file Data_Sheet_1.PDF]

A ***MAPT***

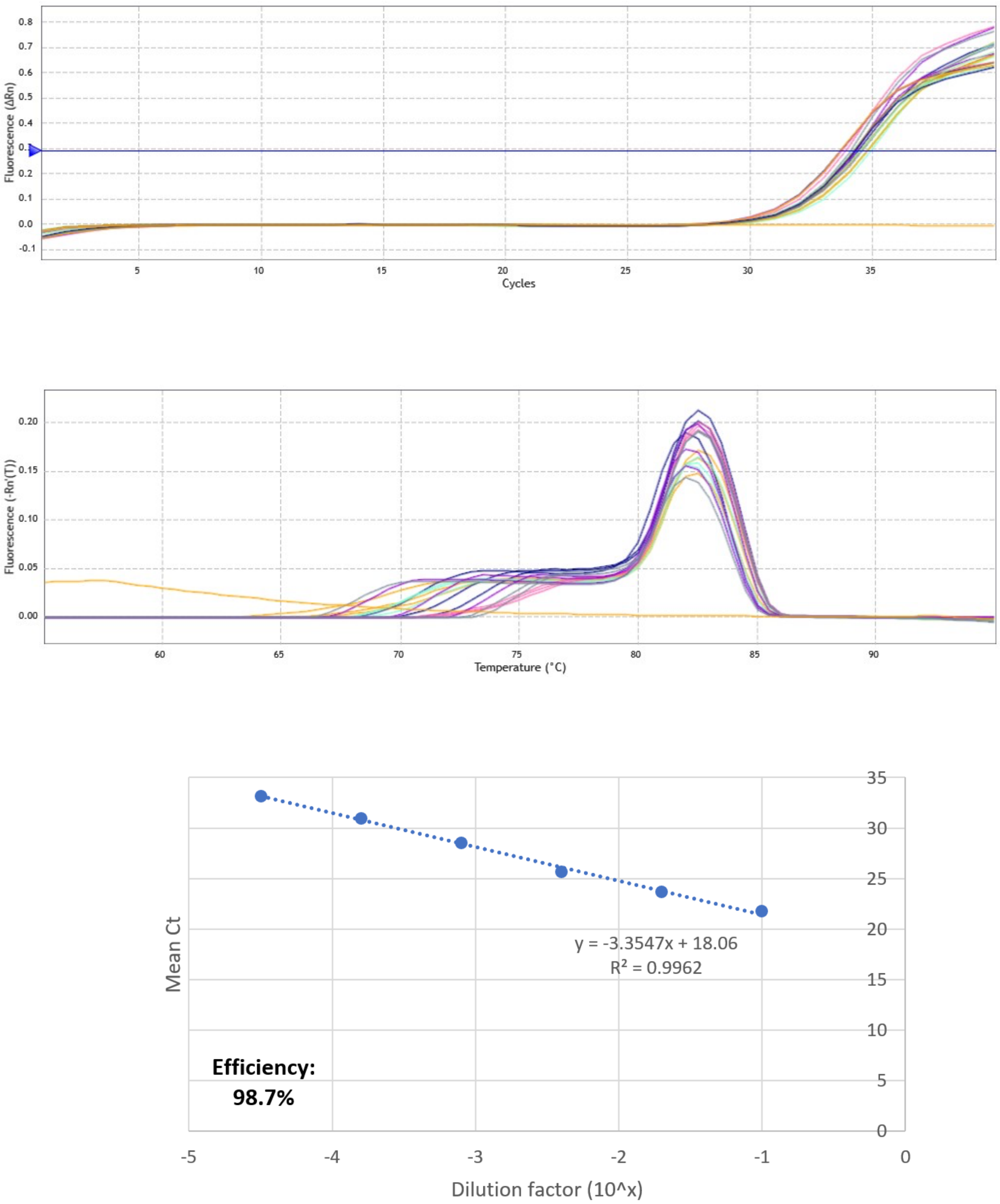

Supplementary Figure 1. Legend page 6.

B *ARL17B*

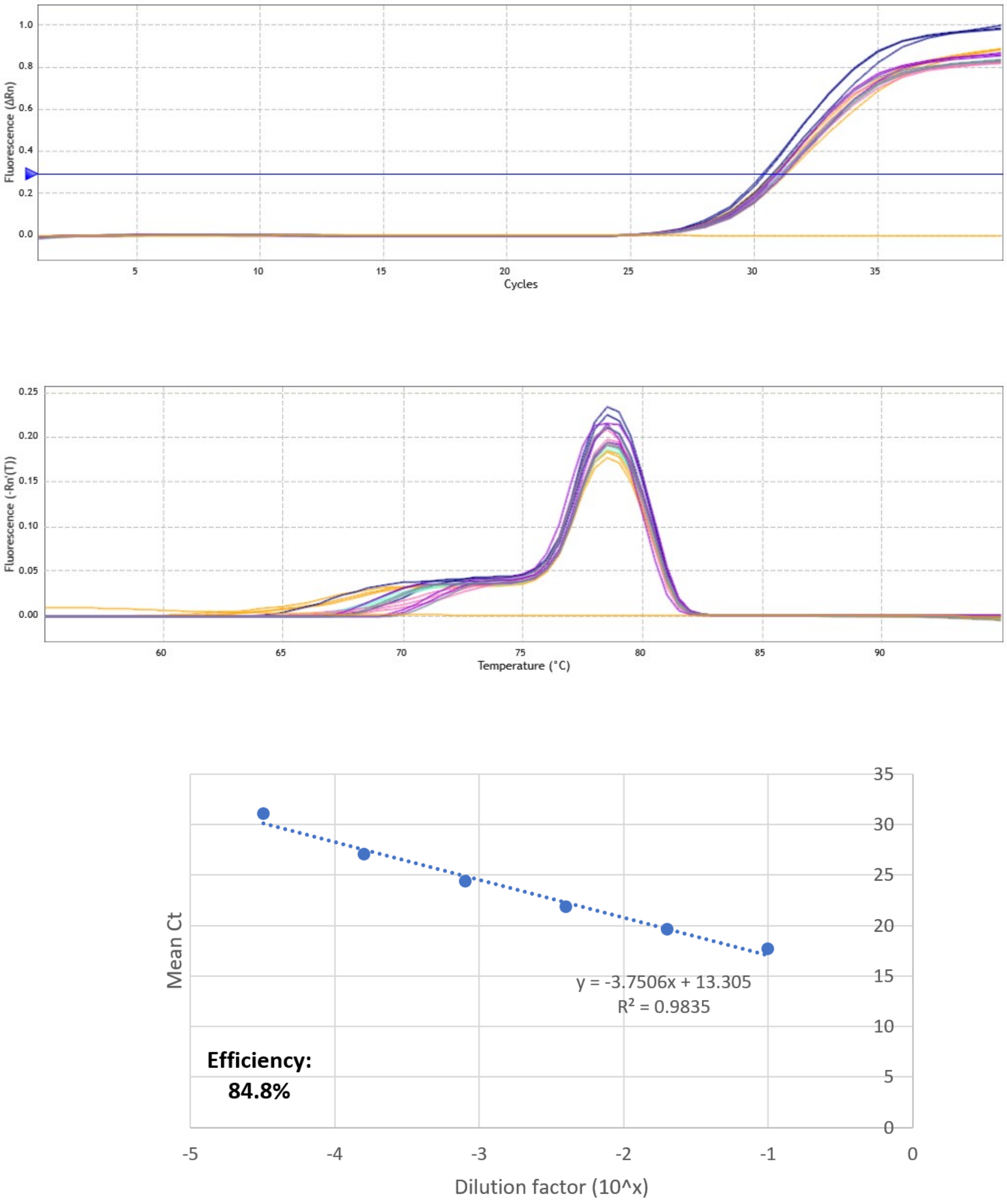

Supplementary Figure 1. Legend page 6.

C ***β-Actin***

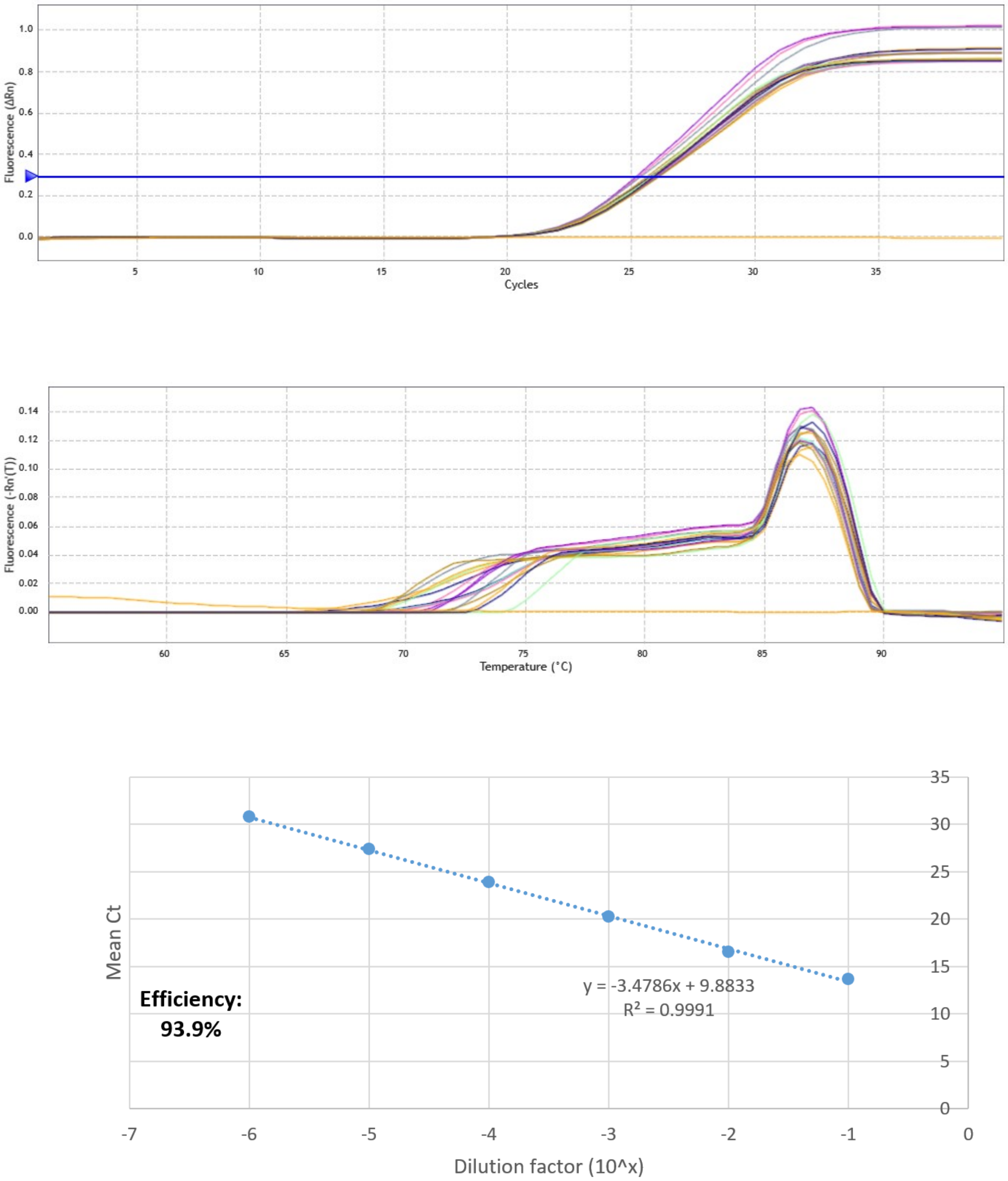

Supplementary Figure 1. Legend page 6.

D ***ARL17A***

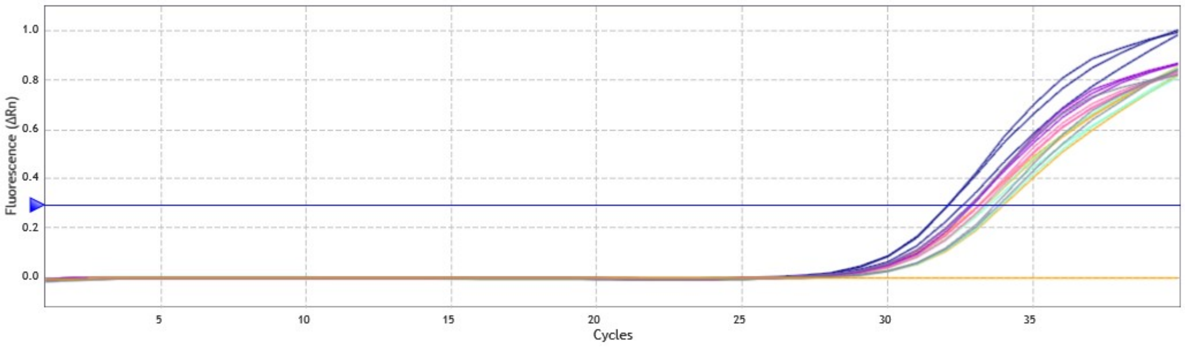

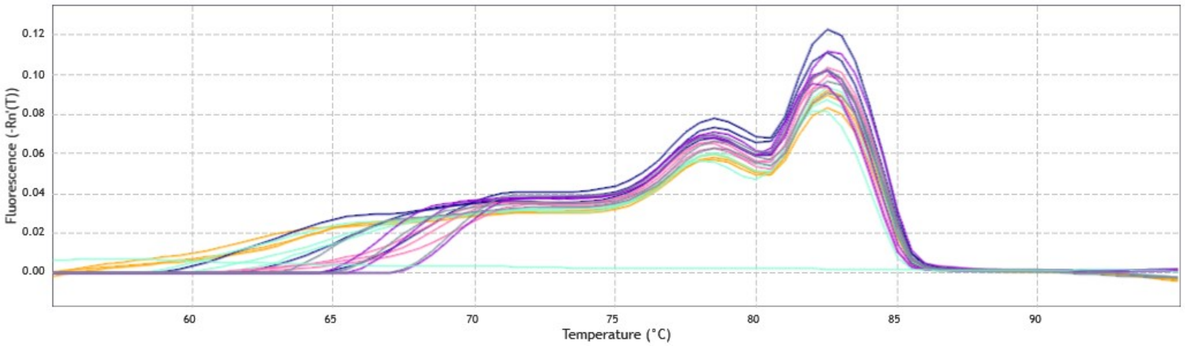

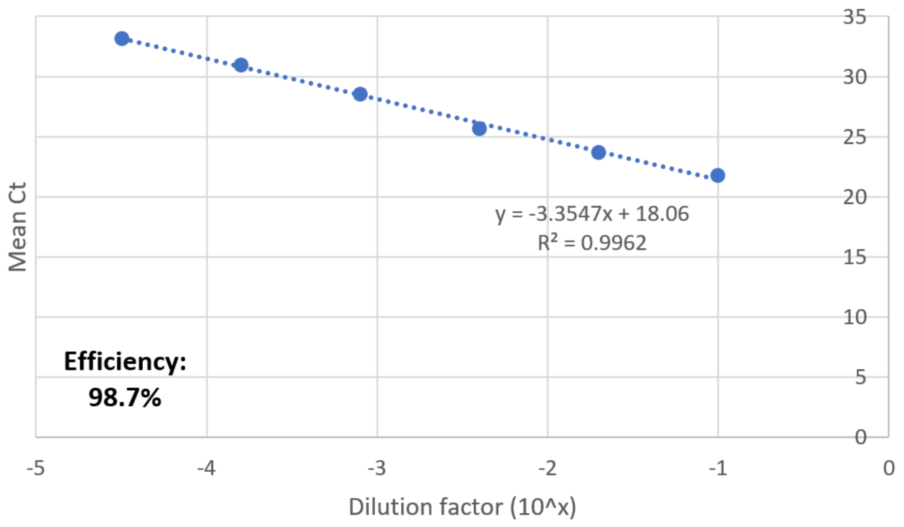

Supplementary Figure 1. Legend page 6.

E ***LRRC37A***

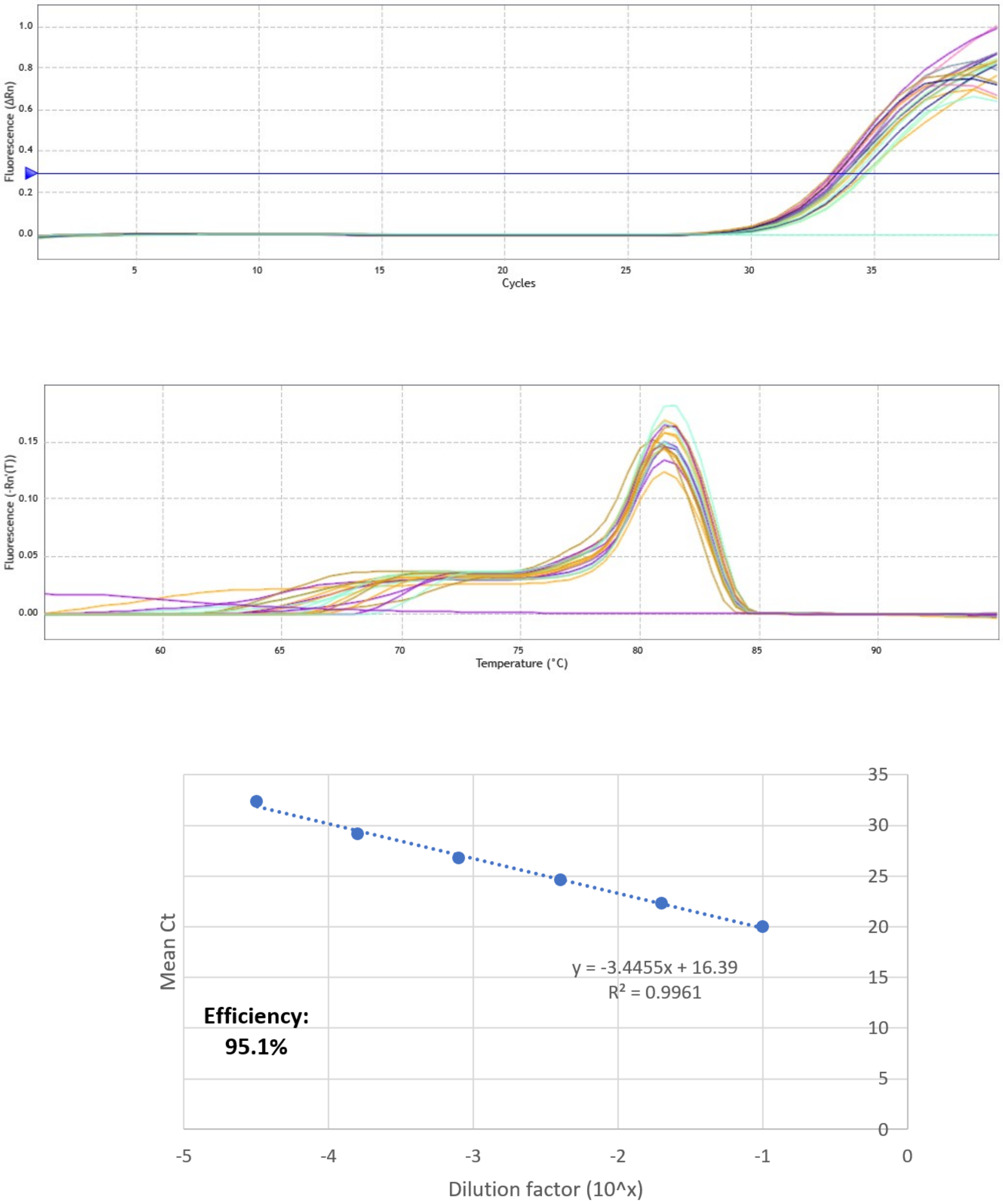

Supplementary Figure 1. Legend page 6.

F ***KANSL1***

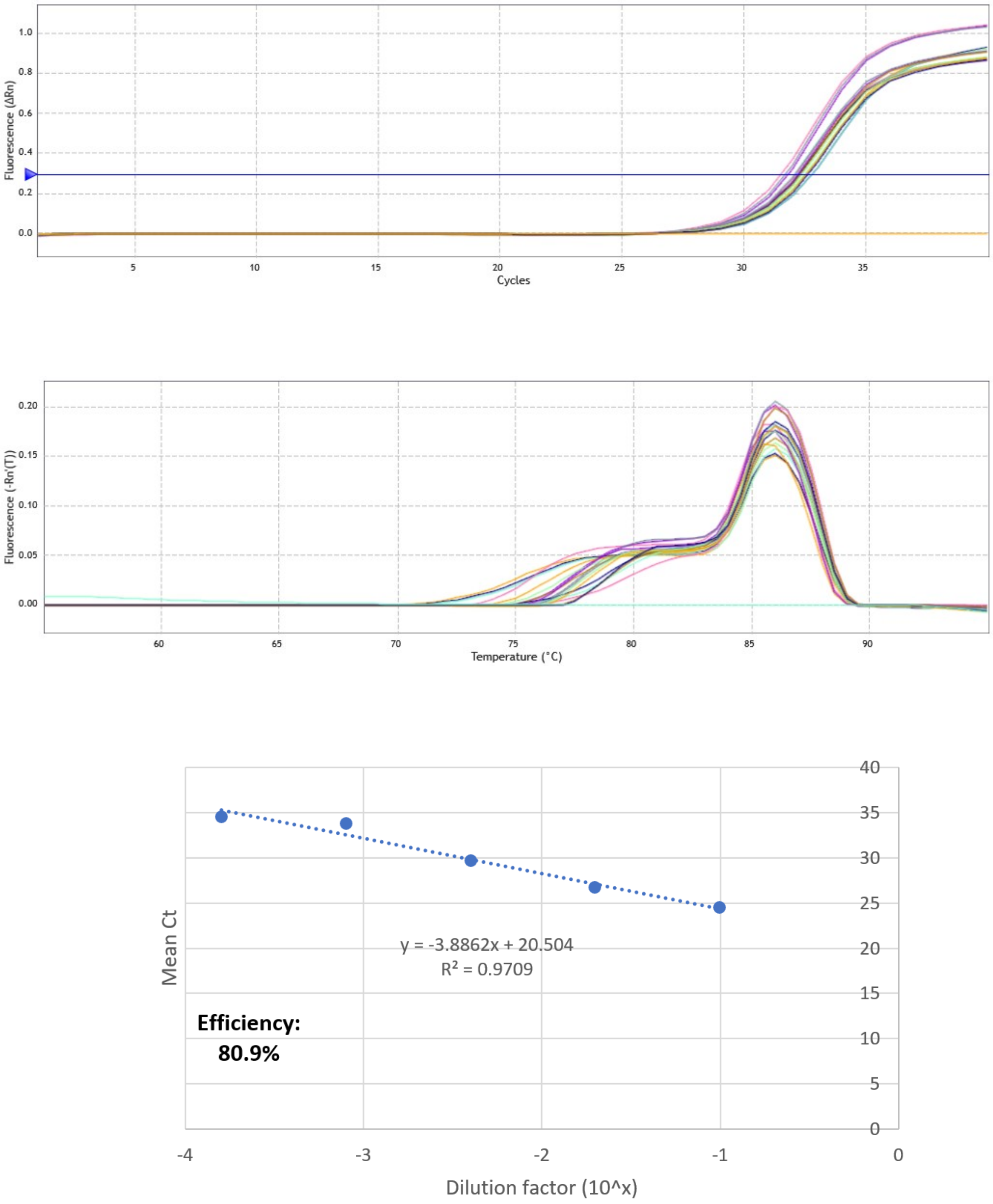

Supplementary Figure 1. Quality control for qPCR validation. Amplification and melting curves showing real time detection of fluorescence (y-axis) versus cycle number (x-axis) for each gene are shown. Non-template controls are included displaying no amplification. In addition, standard curves derived from serial cDNA dilutions as template were generated to assess primer efficiencies. Target genes include *MAPT* (A), *ARL17B* (B), *β-Actin* (C), *ARL17A* (D), *LRRC37A* (E) and *KANSL1* (F).
